# Machine Learning Approach to Predict Emergency Cesarean Sections Among Nulliparous Women

**DOI:** 10.7759/cureus.90501

**Published:** 2025-08-19

**Authors:** Nazanin Rezaei, Masoumeh Amani, Homeira Asgharpoor, Vahid Mehrnoush, Fatemeh Darsareh, Anahita Nikmanesh

**Affiliations:** 1 Obstetrics, Mother and Child Welfare Research Center, Hormozgan University of Medical Sciences, Bandar Abbas, IRN; 2 General Surgery, Mother and Child Welfare Research Center, Hormozgan University of Medical Sciences, Bandar Abbas, IRN; 3 Medicine, Mother and Child Welfare Research Center, Hormozgan University of Medical Sciences, Bandar Abbas, IRN

**Keywords:** artificial intelligence, cesarean section, childbirth, machine learning, mode of delivery, nulliparous women

## Abstract

Introduction

The obstetrical team's efforts are consistently focused on minimizing the number of cesarean sections, particularly in nulliparous women. One of the most crucial steps is to understand the risk factors that predispose the woman to a cesarean section. This study aimed to identify the predictors of emergency cesarean sections in nulliparous women using a machine learning approach.

Methods

A retrospective cohort study was carried out at a maternal tertiary center in Iran among nulliparous women with a single cephalic pregnancy, ≥37 weeks of gestation, and induced or spontaneous labor, who gave birth between January 2020 and December 2022. The exclusion criteria were maternal request for cesarean section or those who delivered via cesarean section before the onset of labor. The rate of emergency cesarean section and the performance of machine learning in predicting emergency cesarean section were the outcome measures. Twenty-three factors potentially linked to the method of childbirth were initially identified, and included age, educational level, place of residence, medical insurance, nationality, attending prenatal education course, gestational age, the onset of labor, having a doula during the labor process, analgesia during labor, history of infertility, history of abortion, maternal anemia, cardiovascular disease, diabetes, maternal obesity, preeclampsia, prolonged rupture of membrane, placenta abruption, meconium amniotic fluid, intrauterine growth retardation, newborn weight, and newborn sex. The input data were fed into seven machine learning models: linear regression, logistic regression, decision tree classification, random forest classification, XGBoost classification, permutation classification (KNN), and deep learning.

Results

During the study period, 1916 (71.8%) of the 2668 births were vaginal, while 752 (28.2%) were by cesarean section. Cesarean sections were more common in mothers of advanced age and with a higher level of education. Attending a prenatal education course was also linked to the method of childbirth. Induced labor was more common in women who had a cesarean section. Those who had a doula were more likely to give birth vaginally. Maternal diabetes, obesity, preeclampsia, thyroid disease, placental abruption, meconium amniotic fluid, and fetal macrosomia were all linked to the method of childbirth. The area under the curve (AUC) for each model turned out to be: linear regression (0.86), XGBoost classification (0.83), logistic regression (0.79), deep learning (0.78), permutation classification (K-Nearest Neighbors or KNN) (0.77), decision tree classification (0.76), and random forest classification (0.72). Linear regression had a better diagnostic performance than other models with the area under the ROC curve (AUROC): 0.86, accuracy: 0.82, precision: 0.79, recall: 0.85, and F1-Score: 0.79). The linear regression model showed that advanced maternal age, advanced maternal education, diabetes, preeclampsia, placenta abruption, hypothyroidism, meconium amniotic fluid, late-term pregnancy, doula support, and attending prenatal courses were predictors of emergency cesarean section in nulliparous women.

Conclusions

Utilizing a clinical database and various machine learning algorithms showed potential in predicting emergency cesarean section. Additional prospective research, including intrapartum clinical characteristics, is essential for improving the accuracy of prediction accuracy.

## Introduction

The mode of birth is becoming one of the most serious issues for obstetricians, health authorities, and mothers. Vaginal birth is a natural, physiological procedure. However, in some cases, a cesarean section may be necessary to safeguard the mother's and the baby's health. In such cases, the underuse of cesarean section contributes to higher maternal and neonatal mortality and morbidity. Establishing an acceptable cesarean section rate and the rate that produces optimal mother and child outcomes is also debatable, according to several obstetric scientific societies [1]. Overuse (i.e., the use of cesarean section without a medical basis) has not shown advantages and may cause harm, such as the increased risk of infection and hemorrhage, and waste of human and financial resources [2,3]. Global cesarean section rates have climbed dramatically over the years, from roughly 7% in 1990 to 21% today, surpassing the WHO's ideal acceptable cesarean section rate of 10%-15% [4]. These patterns are likely to continue expanding over the next decade, with both unmet demands and usage coexisting with the estimated global rate of 29% by 2030 [5]. However, the midwifery team's efforts are consistently focused on minimizing the number of cesarean sections, particularly in nulliparous women [6]. One of the most crucial steps is to understand the risk factors that predispose the woman to cesarean section. Numerous characteristics have been identified as risk factors for emergency cesarean section in nulliparous women, and numerous models have been created to predict the mode of childbirth [7,8]. However, the majority of these investigations employed classic statistical models. This study was designed to discover the predictors of emergency cesarean section in nulliparous women using a machine learning approach.

The machine learning approach has lately contributed to the development of models that can improve diagnostic precision in a variety of obstetric settings [9-11]. Recent machine learning models composed of various algorithms have been proven to provide superior prediction of obstetric outcomes, including shoulder dystocia [12], and vaginal birth after cesarean delivery [13]. The current study's goal was to evaluate the utilization of maternal clinical variables to construct a machine learning model for emergency cesarean in nulliparous women during labor.

## Materials and methods

This study employed a retrospective cohort study design. Through literature review [9,11,14], 23 factors that may be associated with the method of childbirth were identified. These included age, educational level, place of residence place, medical insurance, nationality, attending prenatal education course, gestational age, the onset of labor, having a doula during the labor process, analgesia during labor, history of infertility, history of abortion, maternal anemia, cardiovascular disease, diabetes, maternal obesity, preeclampsia, prolonged rupture of membrane, placenta abruption, meconium amniotic fluid, intrauterine growth retardation, newborn weight, and newborn sex and were classified as variables. The data presented above were obtained from the electronic health record system of Khaleej-e-Fars Hospital, a tertiary-care medical center in Bandar Abbas, Iran. As a part of routine clinical care, the midwives gather and maintain electronic health data for all deliveries. We received the electronic health records for 2668 singleton vaginal deliveries between January 2020 and December 2022.

The inclusion criteria were nulliparous women with a single cephalic pregnancy, ≥37 weeks of gestation, and induced or spontaneous labor. The exclusion criteria were maternal request for cesarean section or those who delivered via cesarean section before the onset of labor. The women were divided into two groups: vaginal deliveries and cesarean sections.

The study was approved by the Research Ethics Committees of the Hormozgan University of Medical Sciences (approval no. IR.HUMS.REC.1403.245).

Development and validation of machine learning models

The first part of the analysis was comparing the two groups of women based on all of the variables listed above. The variables with a significant p-value (less than 0.5) were chosen as feature selections for the machine learning method.

Selecting a machine learning algorithm can be a complex task due to the hundreds of options available. In the realm of supervised machine learning, models are developed to forecast either discrete outcomes, known as classification, or continuous outcomes, referred to as regression. The input data were fed into seven machine learning models: linear regression, logistic regression, decision tree classification, random forest classification, XGBoost classification, permutation classification (K-Nearest Neighbors or KNN), and deep learning. Except for the tree-based models, all machine learning models underwent L2 normalization for feature normalization. Each model's outcome ranged between 0 and 1.

A random number generator was used to randomly assign the 2668 demonstrations to either the "training set" (70%) or the "test set" (30%). There is a danger of model overfitting in prediction modeling, where a prediction model is hypersensitive to noise within the training data, and performs significantly better on known training samples while failing to generalize its results on new testing samples. In the training and test sets, the rates of vaginal deliveries and cesarean sections remained unchanged from the original dataset. We utilized the training set to set the parameters for the prediction models, and the "test set" to evaluate how well they worked. We used 10-fold repeated k-fold cross-validation since our training dataset was large. The entire partitioning, training, and validation procedure was repeated a specified number of times, which made repeated cross-validation a simple yet robust extension of traditional cross-validation. The number k=10 was chosen to improve the model performance estimations while still fully exploiting the collected data for the analysis.

Evaluation of the performance of machine learning models 

We employed the accuracy (the proportion of correct predictions among all the predictions made) and the AUC of the receiver operating characteristic (AUROC), the confusion matrix, precision (the number of predictions from a class that belongs to that class 0, recall (the number of predictions generated for a class based on all of the examples in the dataset 0, and F1-Score (weighted average of the precision and recall, where an F1-Score reaches its best value at 1 and worst score at 0) for the performance evaluation.

Statistical analysis

All statistical analyses were done using IBM SPSS Statistics for Windows, Version 25 (Released 2017; IBM Corp., Armonk, New York, United States) and Python software (version 3.7.0, Python Software Foundation, Wilmington, US).

## Results

Demographic factors associated with the method of childbirth

During the study period, 1916 (71.8%) of the 2668 births at our center were vaginal, while 752 (28.2%) were by cesarean section. Table 1 displays the maternal demographic characteristics associated with the method of childbirth.

**Table 1:** Demographic factors associated with the method of childbirth Data are presented as n (%).

Demographic characteristics	Vaginal delivery (n=1916)	Cesarean section (n=752)	P-value	X^2 ^-value
Age (years)			<0.001	34.1
18-35	1840 (96.0)	656 (87.2)		
Above 35	76 (4.0)	96 (12.8)		
Place of residence			0.487	23.2
Urban	1456 (76.0)	553 (73.4)		
Rural	460 (24.0)	200 (26.6)		
Education			0.004	13.8
Primary	304 (15.8)	124 (16.5)		
High school/Diploma	1124 (58.7)	352 (46.8)		
Advanced	488 (25.5)	276 (36.7)		
Medical Insurance			0.230	9.81
Yes	312 (16.3)	92 (12.1)		
No	1604 (83.7)	660 (87.8)		
Prenatal education course			0.003	8.48
Yes	1756 (91.6)	736 (97.9)		
No	160 (8.4)	16 (2.1)		
Nationality			0.297	25.1
Iranian	1880 (98.1)	748 (99.5)		
Non-Iranian	36 (1.9)	4 (0.5)		

Cesarean section was more common in mothers of advanced age and with a higher level of education. Attending a prenatal education course was also linked to the method of childbirth.

Obstetrical factors associated with the method of childbirth

Table 2 depicts the link between birthing methods and obstetrical variables.

**Table 2:** Obstetrical factors associated with the method of childbirth Data are presented as n (%).

Variables	Vaginal delivery (n=1916)	Cesarean section (n=752)	P-value	X^2 ^-value
History of infertility			0.658	8.14
Yes	24 (1.3)	12 (1.6)		
No	1892 (98.7)	740 (98.4)		
History of abortion			0.098	12.3
Yes	36 (1.9)	32 (4.3)		
No	1880 (98.1)	720 (95.7)		
Onset of labor			<0.001	84.7
Spontaneous	1072 (55.9)	288 (38.3)		
Induced	844 (44.1)	464 (61.7)		
Gestational age (weeks)			0.013	31.2
Late-term (more than 41)	332 (17.3)	164 (21.8)		
Term (37-41)	1584 (82.7)	588 (78.2)		
Doula in attendance			<0.001	114.4
Yes	968 (50.5)	44 (5.9)		
No	948 (49.5)	708 (94.1)		
Analgesia during labor				
Yes	624 (32.6)	240 (31.9)	0.621	24.3
No	1292 (67.4)	512 (68.1)		

Induced labor was more common in women who had a cesarean section. Those who had a doula were more likely to give birth vaginally.

Maternal and neonatal clinical factors associated with the method of childbirth

Table 3 depicts the association between maternal and newborn clinical variables and the method of birth.

**Table 3:** Maternal and neonatal clinical factors associated with the method of childbirth Data are presented as n (%).

Variables	Vaginal delivery (n=1916)	Cesarean section (n=752)	P-value	X^2 ^-value
Maternal anemia			0.576	11.9
No	1864 (97.3)	740 (98.4)		
Yes	52 (2.7)	12 (1.6)		
Prolonged rupture of membrane			0.495	7.41
No	1840 (96.0)	732 (97.3)		
Yes	76 (4.0)	20 (2.7)		
Diabetes			0.007	23.12
No	1660 (86.6)	584 (77.7)		
Yes	256 (13.4)	168 (22.3)		
Maternal obesity			<0.001	19.12
No	1584 (82.7)	464 (61.7)		
Yes	332 (17.3)	288 (38.3)		
Preeclampsia			0.009	13.8
No	1872 (97.7)	700 (93.1)		
Yes	44 (2.3)	52 (6.9)		
Thyroid disease			0.003	12.9
No	1708 (89.1)	620 (82.4)		
Yes	208 (10.9)	132 (17.6)		
Placenta abruption			0.021	21.1
No	1908 (99.6)	732 (97.3)		
Yes	8 (0.4)	20 (2.7)		
Meconium			<0.001	23.8
No	1780 (92.9)	556 (73.9)		
Yes	136 (7.1)	196 (26.1)		
Intrauterine growth retardation			0.497	12.4
No	1836 (95.8)	732 (97.3)		
Yes	80 (4.2)	20 (2.7)		
Fetal macrosomia			0.020	15.1
No	1900 (99.2)	720 (95.7)		
Yes	16 (0.8)	32 (4.3)		
Newborn sex			0.923	8.92
Male	968 (50.5)	384 (51.1)		
Female	948 (49.5)	368 (48.9)		

Maternal diabetes, obesity, preeclampsia, thyroid disease, placental abruption, meconium amniotic fluid, and fetal macrosomia were all linked to the method of childbirth.

Performance of the machine learning models in predicting emergency cesarean section

The AUC for each model was linear regression (0.86), XGBoost classification (0.83), logistic regression (0.79), deep learning (0.78), permutation classification - KNN (0.77), decision tree classification (0.76), and random forest classification (0.72). The machine learning models demonstrated varying performance, as indicated in Table 4.

**Table 4:** The performance of machine learning models in predicting emergency cesarean section AUC: The area under the curve; KNN: K-Nearest Neighbors

Machine learning model	Accuracy	Precision	Recall	F1-Score	AUC
Linear regression	0.82	0.79	0.85	0.79	0.86
XGBoost classification	0.77	0.73	0.80	0.79	0.83
Logistic regression	0.78	0.75	0.81	0.77	0.79
Deep learning	0.77	0.73	0.80	0.77	0.78
Permutation classification - KNN	0.75	0.71	0.80	0.75	0.77
Decision tree classification	0.75	0.73	0.78	0.75	0.76
Random forest classification	0.69	0.62	0.73	0.79	0.72

Linear regression had a better diagnostic performance among all the models with AUROC: 0.86, accuracy: 0.82, precision: 0.79, recall: 0.85, and F1-Score: 0.79).

Predictors of emergency cesarean section in nulliparous women

**Figure 1:**
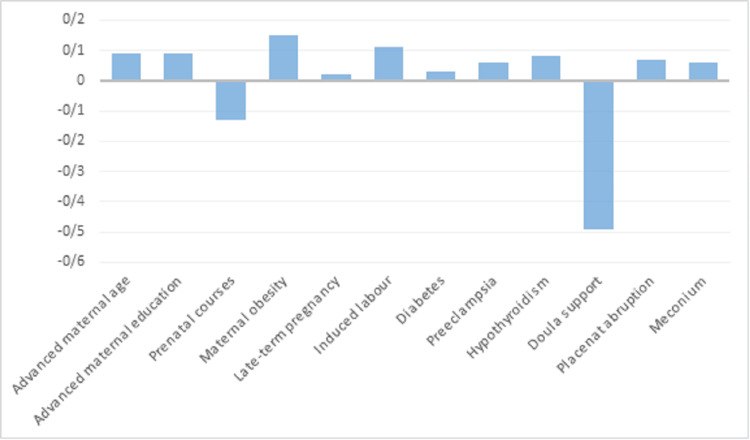
Feature importance of the linear regression algorithm in the prediction of cesarean section

According to the linear regression model that demonstrated the highest effectiveness among the models studied, factors such as older maternal age, higher maternal education, diabetes, preeclampsia, placental abruption, hypothyroidism, meconium-stained amniotic fluid, late-term pregnancies, assistance of a doula, and participation in prenatal classes were identified as predictors of emergency cesarean sections in nulliparous women. As illustrated in Figure 1, participating in birth classes and receiving assistance from a doula led to fewer emergency cesarean deliveries.

## Discussion

During the study period, 1916 (71.8%) of the 2668 births in our center were vaginal, while 752 (28.2%) were by cesarean section. The AUC for each model turned out to be: linear regression (0.86), XGBoost classification (0.83), logistic regression (0.79), deep learning (0.78), permutation classification - KNN (0.77), decision tree classification (0.76), and random forest classification (0.72). Linear regression had a better diagnostic performance among the models with the AUROC curve: 0.86, accuracy: 0.82, precision: 0.79, recall: 0.85, and F1-Score: 0.79. The linear regression model showed that advanced maternal age, advanced maternal education, diabetes, preeclampsia, placenta abruption, hypothyroidism, meconium amniotic fluid, late-term pregnancy, doula support, and attending prenatal courses were the predictors of emergency cesarean section in nulliparous women.

The recommended cesarean rate for nulliparous women with a single cephalic pregnancy at 37 weeks of gestation or more in spontaneous or induced labor is less than 10% [15], while the incidence in our research sample was 28.2%. The rising cesarean section rate in nulliparous women is a global concern because the first cesarean can lead to subsequent cesareans, which ultimately increases their overall rate [16]. The reasons for the increase in cesarean sections are numerous, but extant literature suggests that it is primarily due to advanced maternal age, particularly in nulliparous women [17,18]. According to our findings, maternal age was associated with the method of childbirth. A higher rate of cesarean section was observed among women equal to or greater than 35 years of age. Social, educational, and demographic developments have resulted in an increasing percentage of women delaying their pregnancies till later in their fertile lives. This social tendency, combined with greater access to birth control, has increased the number of women who become pregnant after the age of 35 [19].

Education is an important socioeconomic factor of health and is usually regarded to be associated with pregnancy outcomes [20]. According to our findings, women with greater education were at a higher risk of giving birth via a cesarean section. Our findings contradict earlier research that demonstrated that greater levels of education were associated with a protective impact, lowering the likelihood of cesarean section [21]. These disparities could be explained by the fact that formal education may not have a direct correlation with health literacy. We suggest that, due to this disparity, it is preferable to investigate the level of health literacy in the manner of birthing rather than schooling. Prenatal education courses are one of the most effective ways to educate pregnant women and raise their health literacy about the birthing process. According to the literature, attending a prenatal education course dramatically lowered the incidence of cesarean sections [22,23]. Our findings also indicate that a prenatal education course had a protective effect against cesarean deliveries.

In line with prior studies [24,25], women who had a doula supporting them during labor had a decreased rate of cesarean section. A doula can provide reassurance, empathy, and a calming presence and can help to alleviate the worry and fear that may arise during a surgical birth.

Among obstetrical factors, gestational age, and the onset of labor were linked to an increased rate of cesarean sections. Post-term and late-term pregnancies were connected with an increased risk of cesarean section [26]. This could be explained by the fact that increasing gestational age may increase the newborn weight and meconium amniotic fluid, both of which have been identified as risk factors for a cesarean section.

Based on our findings, women whose labor was induced were more likely to undergo cesarean delivery than those who began labor spontaneously. The most common indications for inducing the patients are preeclampsia and diabetes, both of which have been established as risk factors for cesarean section. According to a recent study, induction of labor in medically uncomplicated nulliparous women at term more than doubles the incidence of emergency cesarean section compared to spontaneous labor [27].

In terms of maternal comorbidities, the method of childbirth was associated with maternal obesity, diabetes, preeclampsia, hypothyroidism, meconium amniotic fluid, and placenta abruption during labor. According to our findings, multiple studies have found that increasing maternal BMI was connected with an increased emergency cesarean section rate [28,29]. The pathophysiological explanation for the increased cesarean section rates is that an increased BMI, caused by hormonally active adipose tissue, may predispose women to a reduced response to induced labor due to altered metabolic status when overweight or obese [30]. Furthermore, some of the purported effect on increased cesarean section rates is probably due to the higher prevalence of comorbidities in women with a high BMI [31].

A rise in the number of cesarean sections in diabetic mothers was not unexpected. Diabetic women are more likely to have fetal macrosomia [32], which is one of the leading causes of cesarean delivery. Furthermore, terminating the pregnancy in the 39th week, when the woman may not have a suitable Bishop score, can result in prolonged labor and an increased cesarean section [33].

Several studies have reported an increased rate of cesarean section due to preeclampsia [34,35]. This rise could be attributed to concerns about the mother's deterioration and resistant blood pressure, which can be recommended in cases where vaginal deliveries are expected to be delayed.

According to our findings, women who had hypothyroidism documented in their medical records were more likely to have a cesarean section. Previous research suggested that even treated hypothyroidism could result in an increased incidence of cesarean sections [36]. A normal thyroid function is required to maintain normal energy and lipid metabolism [37]. Weight gain or lipid abnormalities associated with hypothyroidism may increase the risk of poor pregnancy outcomes. Changes in the energy metabolism and weight may explain the apparent link between hypothyroidism and gestational diabetes, which has been identified as a significant risk factor for a cesarean section [38].

Placenta abruption during labor is usually considered an obstetric emergency because of the heightened risk to the fetus, necessitating immediate delivery and, in most circumstances, a cesarean section [39]. A cesarean section was reported to be more common in pregnancies with meconium-stained amniotic fluid. The higher risk of fetal distress in situations with amniotic fluid stained with meconium justifies the increased use of an emergency cesarean section [40].

As stated previously, the first stage of data analysis involved determining the risk variables for emergency cesarean delivery in nulliparous women. The next stage was to evaluate the effectiveness of seven machine learning models in predicting emergency cesarean sections. Out of the seven machine learning models, the linear regression model had the best performance for predicting emergency cesarean sections among nulliparous women. The overall accuracy of the linear regression model was 0.82, with an AUC of 0.86. Wie et al. conducted a previous study that employed clinical data from pregnancies and nine machine learning algorithms to predict emergency cesarean deliveries in 1391 term nulliparous women. According to them, the logistic regression model with an overall accuracy of 0.78 performed the best [41]. More studies with larger samples are needed to reach a better conclusion.

Identifying the factors that place women at risk for an emergency cesarean section may help the obstetrics team in providing better consultation during prenatal care in order to minimize these cesarean sections, particularly in nulliparous women. More research should be conducted to analyze appropriate variables and develop big data to determine the best model.

This study's strength lies in its use of a clinical database and multiple machine learning algorithms. We took steps to minimize selection bias by ensuring the inclusion of all mothers who gave birth consecutively during the study period. We could only examine the model's performance after childbirth, not before or during labor which is a limitation of the study.

## Conclusions

Based on the findings, cesarean sections were more common in mothers of advanced age and with a higher level of education. Attending a prenatal education course was also linked to the method of childbirth. Induced labor was more common in women who had a cesarean section. Those who had a doula were more likely to give birth vaginally. The linear regression model showed that advanced maternal age, advanced maternal education, diabetes, preeclampsia, placenta abruption, hypothyroidism, meconium amniotic fluid, late-term pregnancy, doula support, and attending prenatal courses were predictors of emergency cesarean sections in nulliparous women. The rising cesarean section rate among nulliparous women is a global concern, as the first cesarean might lead to repeated cesareans, increasing their overall rate. Using a clinical dataset and numerous machine learning models to help determine the type of delivery seems promising. If verified in additional groups, the prediction system could be used to provide tailored counseling to nulliparous women. More prospective studies with intrapartum clinical and ultrasonographic characteristics are required to improve prediction performance.
